# Non-Canonical Role of IKKα in the Regulation of STAT1 Phosphorylation in Antiviral Signaling

**DOI:** 10.1371/journal.pone.0168696

**Published:** 2016-12-19

**Authors:** Fei Xing, Tomoh Matsumiya, Yuko Shiba, Ryo Hayakari, Hidemi Yoshida, Tadaatsu Imaizumi

**Affiliations:** Department of Vascular Biology, Institute of Brain Science, Hirosaki University Graduate School of Medicine, Hirosaki, Japan; Karolinska Institutet, SWEDEN

## Abstract

Non-self RNA is recognized by retinoic acid-inducible gene-I (RIG-I)-like receptors (RLRs), inducing type I interferons (IFNs). Type I IFN promotes the expression of IFN-stimulated genes (ISGs), which requires the activation of signal transducer and activator of transcription-1 (STAT1). We previously reported that dsRNA induced STAT1 phosphorylation via a type I IFN-independent pathway in addition to the well-known type I IFN-dependent pathway. IκB kinase α (IKKα) is involved in antiviral signaling induced by dsRNA; however, its role is incompletely understood. Here, we explored the function of IKKα in RLR-mediated STAT1 phosphorylation. Silencing of IKKα markedly decreased the level of IFN-β and STAT1 phosphorylation inHeH response to dsRNA. However, the inhibition of IKKα did not alter the RLR signaling-mediated dimerization of interferon responsive factor 3 (IRF3) or the nuclear translocation of nuclear factor-κB (NFκB). These results suggest a non-canonical role of IKKα in RLR signaling. Furthermore, phosphorylation of STAT1 was suppressed by IKKα knockdown in cells treated with a specific neutralizing antibody for the type I IFN receptor (IFNAR) and in IFNAR-deficient cells. Collectively, the dual regulation of STAT1 by IKKα in antiviral signaling suggests a role for IKKα in the fine-tuning of antiviral signaling in response to non-self RNA.

## Introduction

Microorganism invasion in a vertebrate host is initially recognized by pattern recognition receptors (PRRs), resulting in the activation of the innate immune system [1]. RIG-I-like receptors (RLRs) are PRRs that recognize non-self RNA in the cytoplasm. Following the recognition of non-self RNA, RIG-I undergoes a conformational change to interact with a downstream adaptor molecule, mitochondrial antiviral signaling protein (MAVS) [2], which is also known as virus-induced signaling adaptor (VISA) [3], interferon (IFN)-β promoter stimulator-1 (IPS-1) [4], or caspase activation and recruitment domain adaptor inducing IFN-β (Cardif) [5]. Subsequently, MAVS activates downstream signaling molecules to produce type I IFNs [6]. Type I IFNs are essential for mounting a robust host response against viral infection [7]. The type I IFN family predominantly comprises IFN-α and IFN-β, which share a common cell surface receptor that is composed of two chains, the α-chain (IFNAR1) and the β-chain (IFNAR2) [8]. After type I IFN binds to these IFNARs, each subunit of the receptor activates the other (cross-phosphorylation), resulting in subsequent activation of Janus tyrosine kinases (JAK1 and Tyk2) [9]. JAK1 and Tyk2 in turn activate their downstream effectors, signal transducer and activator of transcription (STAT) 1 and STAT2 [7]. The activated STATs form heterodimers to induce the transcription of hundreds of IFN-stimulated genes (ISGs) [10]. Because ISGs are required for the antiviral response [11], STAT1 has a critical role in antiviral innate immunity. STAT1 activation in antiviral signaling is believed to be dependent on type I IFN [12, 13]. We previously showed that double-stranded RNA (dsRNA) induced STAT1 phosphorylation in a type I IFN-independent manner [14]. The regulation of this STAT1 phosphorylation in RLR signaling remains to be elucidated.

Nuclear factor-κB (NFκB) is an important transcription factor that is activated by a variety of stimuli, including cytokines, stress, and pathogenic components [15]. In resting conditions, NFκB is sequestered by inhibitor of NFκB (IκB). IκB is phosphorylated by the IκB kinase (IKK) complex composed of IKKα, IKKβ, and IKKγ (also known as NFκB essential modulator; NEMO), leading to its degradation and the subsequent activation of NFκB [16]. IKKβ and IKKγ are essential for canonical NFκB activation [17]. In addition, IKKγ is required to activate interferon regulatory factor 3 (IRF3) in response to viral infection [18]. However, IKKα is not required for the activation of the canonical NFκB pathway or IRF3, indicating a non-canonical role of IKKα in cellular signal transduction, including antiviral signaling. Indeed, IKKα was shown to be critical in Toll-like receptor (TLR) 7/9 signaling to produce type I IFN [19]. However, the TLR7/9-mediated signaling pathways are considerably different from RLR signaling; thus, the molecular role of IKKα in RLR signaling remains to be elucidated.

In this study, we investigated the role of IKKα in RLR signaling, which is essential for antiviral innate immune responses in non-professional antigen-presenting cells, such as epithelial cells. We unexpectedly found a critical role of IKKα in type I IFN-independent STAT1 activation in RLR signaling. Our data reveal that IKKα contributes to both type I IFN-dependent and independent STAT1 activation in RLR signaling. These findings demonstrate a novel role of IKKα in the antiviral innate immune system.

## Materials and Methods

### Cell Culture

HeLa cells were obtained from the Japanese Collection of Research Bioresources Cell Bank (JCRB, Japan). Human IFNAR-deficient U5A cells and their parental 2fTGH cells were kindly provided by G. Stark (Cleveland Clinic Foundation Research Institute). The cells were maintained in a 5% CO_2_ atmosphere at 37°C in Dulbecco’s modified Eagle’s medium (DMEM) (Sigma-Aldrich, St. Louis, MO) supplemented with 10% fetal bovine serum (FBS) (Perbio Science, Switzerland) and antibiotics (Life Technologies, Carlsbad, CA). The cells were treated with an IKK inhibitor (Merck Millipore, Darmstadt, Germany) when indicated.

### Plasmids and siRNA

cDNA encoding full-length IKKα was amplified from HeLa cell cDNA using Phusion DNA polymerase (Finnzymes, Keilaranta, Finland) and the primers SalI-IKKα-F (5'-ATTCGgtcgacCATGGAGCGGCCCCCGGGGCT-3') and NotI-IKKα-R (5'-TTCgcggccgcTCATTCTGTTAACCAACTCCA-3'). The amplified product was inserted into the SalI and NotI sites of a mammalian expression vector, pCMV-HA (Clontech, Mountain View, CA). The DNA construct was analyzed by DNA sequencing. Plasmid DNA was purified using a plasmid purification column (Qiagen, Hilden, Germany).

To obtain the S176A mutant IKKα expression vector, IKKα S176A cDNA was generated using the overlapping PCR method. Two truncated IKKα fragments were separately amplified by the primer pairs SalI-IKKα-F and 5'-R (5'-AGCTCTGGGGCCAGATACTG-3') or 3'-R (5'-TGATCAAGGAgcTCTGTGT-3'), including the mutant bases, and NotI-IKKα-R. Then, the full-length IKKα S176A cDNA was generated by PCR using the primer pair SalI-IKKα-F and NotI-IKKα-R, and the two IKKα fragments were simultaneously used as the templates. The full-length IKKα S176A was cloned into the SalI-NotI site of pBlueScript II SK(+). Following confirmation of the DNA sequences, the full-length IKKα S176A was transferred to pCMV-HA. The S180A mutant IKKα expression vector was generated by site-directed mutagenesis using the IKKα-encoding vector pBlueScript II SK(+) as the template as well as the primer pair S180A-sense (5'-TCTGTGTACAgCTTTTGTGGG-3') and S180A-antisense (5'-CCCACAAAAGcTGTACACAGA-3'). Then, the SalI-NotI cDNA fragment in the pBlueScript II SK(+)-IKKα-S180A vector was cloned into pCMV-HA.

siRNAs targeting IKKα (SI02654659) and a non-silencing control siRNA were purchased from Qiagen. siRNAs targeting IKKβ (s7265) and IKKγ (s16186) were purchased from Life Technologies.

### Transfection

Transient transfections of HeLa and U5A cells were performed as previously described [14]. Briefly, the cells were seeded at a density of 1.5×10^5^ or 2.5×10^5^ cells per well, respectively, in 12-well culture plates for 16 to 20 h prior to transfection and grown to 70–80% confluency. The cells were transfected with a pCMV-HA-IKKα vector using polyethylenimine (PEI) (Polysciences, Inc., Warrington, PA) and incubated for 24 h for overexpression of exogenous IKKα. HeLa cells or U5A cells were transfected with a non-self dsRNA analog (poly I:C) using TransFectin (Bio-Rad, Hercules, CA) for the indicated period of time. RNA interference (RNAi) was performed via transfection with gene-specific siRNAs or control siRNA using Lipofectamine RNAiMAX (Life Technologies), following the manufacturer’s instructions.

### Quantitative RT-PCR

Total RNA was extracted from the cells using an Illustra RNAspin Mini RNA Isolation Kit (GE Healthcare, Piscataway, NJ). Total RNA (500 ng) served as a template for single-stranded cDNA synthesis in a reaction using an oligo(dT)_18_ primer and M-MLV reverse transcriptase (Life Technologies) under the conditions indicated by the manufacturer. A CFX96 real-time PCR detection system (Bio-Rad) was used for the quantitative analyses of IFN-β and 18S rRNA. The sequences of the primers were as follows:

IFN-β-F (5'-ACTGCCTCAAGGACAGGATG-3'),

IFN-β-R (5'-AGCCAGGAGGTTCTCAACAA-3'),

IL6-F (5'-AGGAGACTTGCCTGGTGAAA-3'),

IL6-R (5'-CAGGGGTGGTTATTGCATCT-3'),

18S rRNA-F (5'-ACTCAACACGGGAAACCTCA-3'), and

18S rRNA-R (5'-AACCAGACAAATCGCTCCAC-3').

The amplification reactions were performed with SsoAdvanced^TM^ Universal SYBR Green Supermix (Bio-Rad) according to the manufacturer’s specifications. The amplification conditions were as follows: 30 s at 98°C, followed by heating consecutively at 98°C and 58°C for 5 s each for 40 cycles. After the amplification was complete, a melting curve was generated by slowly heating from 65°C to 95°C in 0.5°C increments at 5 s per step, with continuous monitoring of the fluorescence. Quantitative analysis of the data was performed using a CFX manager (Bio-Rad).

### ELISA

Conditioned culture medium was collected at the indicated times and was centrifuged at 12,000 x *g* for 5 min at 4°C to remove cell debris. The IFN-β and IL6 concentrations in the culture medium were then measured using a human IFN-β ELISA kit (Kamakura Techno-Science, Japan) or a Quantikine Human IL6 ELISA kit (R&D Systems, Minneapolis, MN), respectively.

### Immunoprecipitation

Cells were lysed in lysis buffer [10 mM Tris (pH 7.4), 100 mM NaCl, 1.5 mM MgCl_2_, 0.5% NP-40] containing 0.2% protease inhibitors. The lysates were centrifuged at 12,000 rpm for 5 min at 4°C to remove cell debris. Protein G Dynabeads (Life Technologies) were preincubated with an anti-STAT1 antibody (Santa Cruz Biotechnology, Santa Cruz, CA) or a non-immune control antibody (Santa Cruz Biotechnology) for 10 min at room temperature, followed by incubation with protein lysates at 4°C for 1 h. After 3 washes with PBS-0.1% Tween 20, the immunoprecipitates were eluted with 3×SDS sample buffer at 95°C for 10 min. Anti-HA antibody beads (Wako, Japan) were incubated with protein lysates overnight at 4°C to pull down exogenous IKKα. After 5 washes using PBS-0.1% Tween 20, the immunoprecipitates were eluted with 3×SDS sample buffer at 95°C for 10 min. Then, the immunoprecipitates were subjected to SDS-PAGE.

### Immunoblot Analyses

After two washes with phosphate-buffered saline (PBS; pH 7.4), cells were lysed in hypotonic lysis buffer [10 mM Tris (pH 7.4), 100 mM NaCl, 1.5 mM MgCl_2_, and 0.5% NP-40] containing 0.2% protease inhibitors. The cell lysates were cleared by centrifugation at 12,000 rpm for 5 min at 4°C, and 10 μg of the lysate was subjected to electrophoresis on a 7.5% SDS-polyacrylamide gel. The proteins were then transferred to polyvinylidene fluoride (PVDF) membranes (Millipore, Billerica, MA), which were subsequently blocked for 1 h at room temperature in TBST buffer [20 mM Tris (pH 7.4), 150 mM NaCl, and 0.1% Tween 20] containing 5% nonfat dry milk (blocking buffer). Next, the membranes were incubated overnight at 4°C with one of the following primary antibodies: rabbit anti-IKKα, rabbit anti-IKKβ, rabbit anti-IKKγ, rabbit anti-JAK1, rabbit anti-Tyk2, rabbit anti-phospho-JAK1, rabbit anti-phospho-Tyk2, rabbit anti-p50, rabbit anti-p52, rabbit anti-Rel B, rabbit anti-IκBα, rabbit anti-phospho-STAT2, and rabbit anti-STAT2 (Cell Signaling), rabbit anti-IRF3 (IBL, Japan), rabbit anti-β-actin (Sigma-Aldrich), mouse anti-HA (Covance), rabbit anti-STAT1, mouse anti-phospho-STAT1, mouse anti-HSP90, mouse anti-p65, or mouse anti-histone (Santa Cruz Biotechnology). After the membranes were washed five times with TBST, they were further incubated for 1 h at room temperature with a bovine anti-rabbit (Santa Cruz Biotechnology) or a ZyMax anti-mouse IgG antibody (Life Technologies) coupled to horseradish peroxidase (HRP) at a 1:10,000 dilution in blocking buffer. The washes were repeated using TBST, and the immunoreactive bands were then visualized using Luminata Crescendo Western HRP Substrate (Millipore).

For native PAGE analysis, the cells were harvested in native lysis buffer [50 mM Tris-HCl (pH 8.0), 1% NP-40, 150 mM NaCl]. After centrifugation at 12,000 rpm for 10 min at 4°C, the lysates were subjected to native PAGE as previously reported [20].

### Nuclear Fractionation

Following cell lysis with hypotonic lysis buffer and centrifugation at 2,000 rpm for 15 min at 4°C, the supernatant was collected (cytoplasmic fraction), and the pellet (nucleus) was resuspended in RIPA buffer and passed through a 26 G needle-attached syringe. Then, the lysate was centrifuged at 12,000 rpm for 10 min at 4°C, and the supernatant (nuclear fraction) was collected. Both the cytoplasmic and nuclear fractions were further subjected to SDS-PAGE.

### Immunofluorescence Analyses

HeLa cells that were grown on glass coverslips were transfected with rhodamine-labeled poly I:C (InvivoGen) and then fixed with 4% formaldehyde for 20 min, permeabilized with 0.1% Triton X-100 for 10 min and blocked with 3% BSA for 1 h. The cells were then incubated for 1 h with mouse monoclonal anti-IKKα (Millipore) and rabbit polyclonal anti-STAT1 (Santa Cruz Biotechnology) antibodies. After a washing step, the cells were incubated with Alexa 488-conjugated anti-mouse IgG and Alexa 555-conjugated anti-rabbit IgG. The cells were mounted in ProLong Gold antifade reagent (Life Technologies), and the subcellular localizations of IKKα and STAT1 were visualized by confocal laser scanning microscopy (C1si, Nikon, Japan).

### Statistics

Statistical significance was analyzed using a one-way ANOVA followed by a post hoc Fisher’s protected least significant difference test. All probability (*P*) values were based on two-tailed tests, and *P* < 0.05 was considered to be significant.

## Results

### Role of IKKα in RLR-mediated antiviral signaling

**Fig 1:**
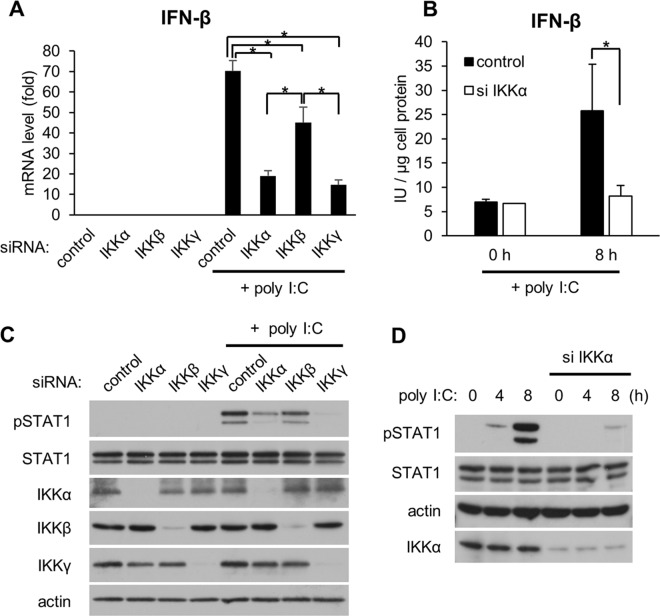
Effects of IKKs on IFN-β expression in response to poly I:C. (A, C) HeLa cells were transfected with siRNA targeting IKKα, IKKβ, and IKKγ or control siRNA. Forty-eight hours after the transfection, the cells were further transfected with poly I:C (50 ng/well) for an additional 4 h. (A) The expression of IFN-β mRNA was analyzed by qRT-PCR. Data are presented as the mean ± SD of three independent experiments. *P<0.01. (B, D) Following IKKα RNAi silencing, the cells were transfected with poly I:C (50 ng/well) for 8 h (B) or up to 8 h (C). (B) IFN-β levels were determined by ELISA. All data are shown as the mean of three independent experiments. *P<0.01. (C, D) Protein levels were analyzed by immunoblotting. The results are representative of three independent experiments.

We initially determined whether IKKα is involved in the expression of IFN-β, a type I IFN produced from a variety of cells, including epithelial cells, in response to activation of RLR signaling. We previously reported that transfection of polyinosinic-polycytidylic acid (poly I:C), a synthetic viral dsRNA analog, activates RLR signaling in HeLa cells [20]. As expected, poly I:C markedly stimulated the expression of IFN-β mRNA and protein (Fig 1A and 1B). IKKγ, not IKKα or IKKβ, was shown to be the most important IKK in the complex for antiviral signaling [18]. Consistent with this report, silencing of IKKγ decreased the expression of IFN-β in response to poly I:C (Fig 1A). We found that silencing of IKKα in addition to IKKγ substantially decreased the expression of IFN-β. Although suppression of IFN-β was also observed following knockdown of IKKβ, this effect was limited compared with those of IKKα and IKKγ silencing. Activation of STAT1 is required to induce ISGs [21]; therefore, we next examined the role of IKKs in the RLR signaling-mediated STAT1 phosphorylation. We found that silencing of IKKγ or IKKα inhibited the phosphorylation of STAT1 in response to poly I:C transfection (Fig 1C). In contrast, this effect was not observed when IKKβ was silenced by RNAi. Thus, we hypothesized that IKKα has an important role in RLR signaling-mediated antiviral innate immunity. To explore the detailed mechanisms by which IKKα regulates RLR signaling, we focused on the effect of IKKα on RLR signaling-meditated STAT1 phosphorylation. Time-dependent phosphorylation of STAT1 induced by poly I:C was notably inhibited by IKKα knockdown (Fig 1D).

**Fig 2:**
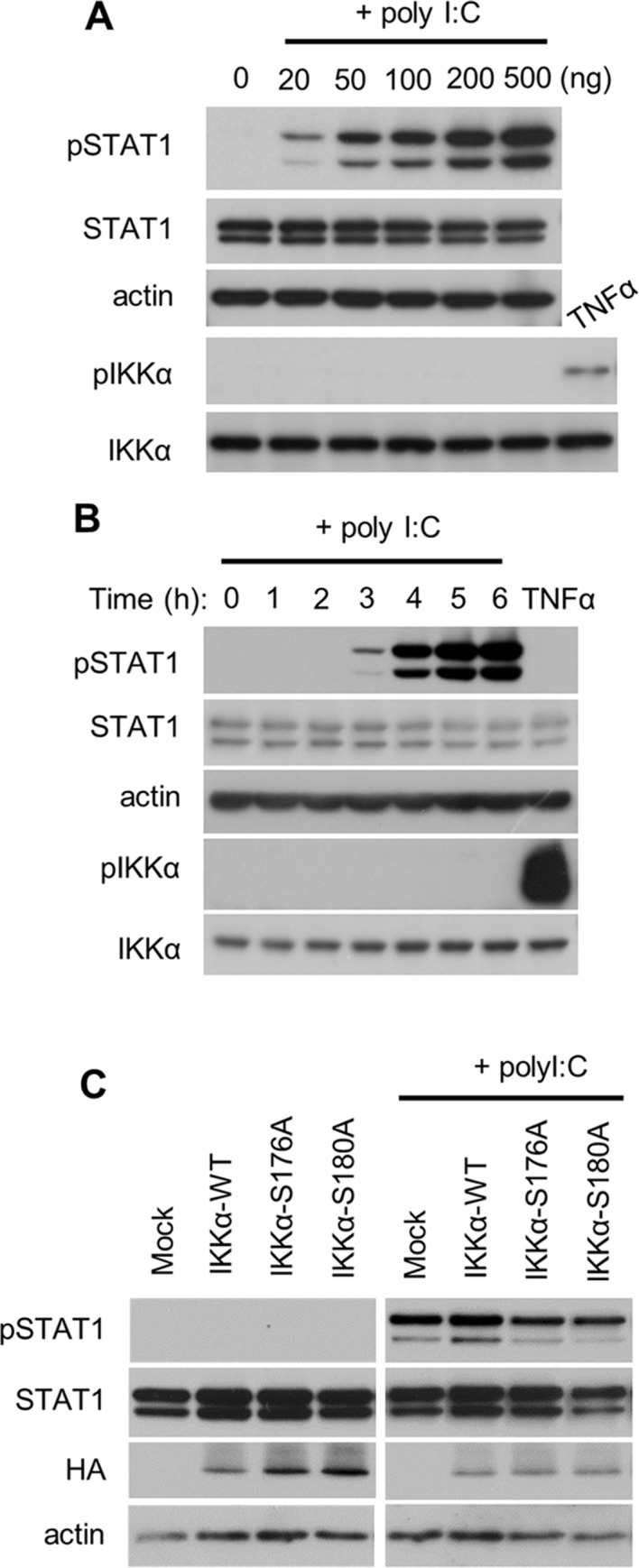
Effects of silencing IKKα on STAT1 phosphorylation in response to dsRNA transfection. (A) HeLa cells were transfected with poly I:C at the indicated concentrations for 4 h. (B) HeLa cells were transfected with poly I:C (50 ng/well) for up to 6 h or treated with TNFα for 10 min. (C) HeLa cells transiently overexpressing WT IKKα, IKKα S176A, or IKKα S180A were transfected with poly I:C for 4 h. Protein levels were analyzed by immunoblotting. The results are representative of three independent experiments.

IKKα is essential for activation of tumor necrosis factor α (TNFα)-medicated intracellular signal transduction [22]. Therefore, we examined whether IKKα was activated when the cells were exposed to dsRNA. Concentration-dependent STAT1 phosphorylation was observed in response to poly I:C (Fig 2A). However, poly I:C failed to stimulate phosphorylation of IKKα, while TNFα rapidly activated IKKα phosphorylation. Furthermore, a time-course experiment showed that poly I:C did not activate IKKα phosphorylation (Fig 2B). The IKKα residues Ser-176 and Ser-180 are essential for activation of downstream signaling, especially for NFκB-mediated signaling [23]; thus, we analyzed the effect of mutations in these residues on STAT1 phosphorylation. Overexpression of wild type (WT) IKKα did not induce STAT1 phosphorylation (Fig 2C), indicating that the amount of IKKα does not affect STAT1 phosphorylation. IKKα mutations at either Ser-176 or Ser-180 did not alter the phosphorylation of STAT1. The levels of poly I:C-induced STAT1 phosphorylation were unchanged by the introduction of exogenous WT IKKα or IKKα mutated at Ser-176 and Ser-180. These results suggest that the phosphorylation of IKKα is not required for RLR-mediated STAT1 activation.

### Inhibition of RLR-mediated STAT1 phosphorylation by an IKK inhibitor

**Fig 3:**
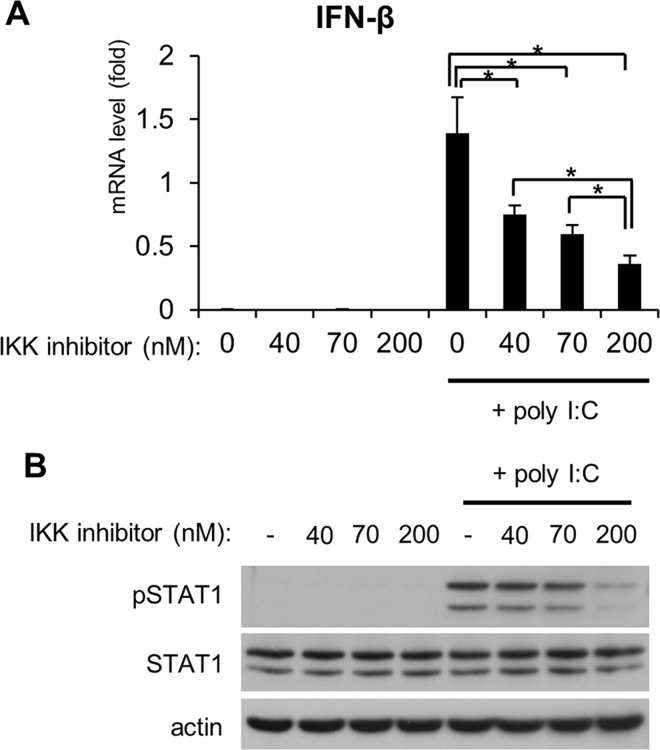
Effect of an IKK inhibitor on STAT1 phosphorylation in response to dsRNA. HeLa cells were pretreated with an IKK inhibitor at the indicated concentrations for 1 h and then transfected with poly I:C (50 ng/well) for 4 h. (A) The expression of IFN-β was analyzed by qRT-PCR. Data are presented as the mean ± SD of three independent experiments. *P<0.01. (B) Cell extracts were subjected to SDS-PAGE and then analyzed by immunoblotting. The results are representative of three independent experiments.

The 2-benzamido-pyrimidines are IKK inhibitors. The IC_50_ analysis for one IKK inhibitor, IKK VII, showed that it inhibited IKKβ activation at 40 nM, formation of the IKK complex at 70 nM, and IKKα activation at 200 nM [24]. When the cells were pretreated with varying concentrations of the IKK inhibitor, poly I:C-induced IFN-β expression was significantly inhibited, even at 40 nM of the IKK inhibitor (Fig 3A). In contrast, no suppression of STAT1 phosphorylation was observed following pretreatment of the cells with 40 nM or 70 nM of the IKK inhibitor (Fig 3B), suggesting a type I IFN- and IKK-independent STAT1 activation pathway in RLR signaling. However, pretreatment of the cells with 200 nM of the IKK inhibitor markedly inhibited the RLR-mediated STAT1 phosphorylation, indicating an IKKα-dependent pathway for STAT1 phosphorylation in RLR signaling. We observed that the IKK inhibitor was active in HeLa cells because it abolished TNFα-induced degradation of IκB in a concentration-dependent manner (S1 Fig).

### Involvement of IKKα in type I IFN-independent STAT1 phosphorylation

**Fig 4:**
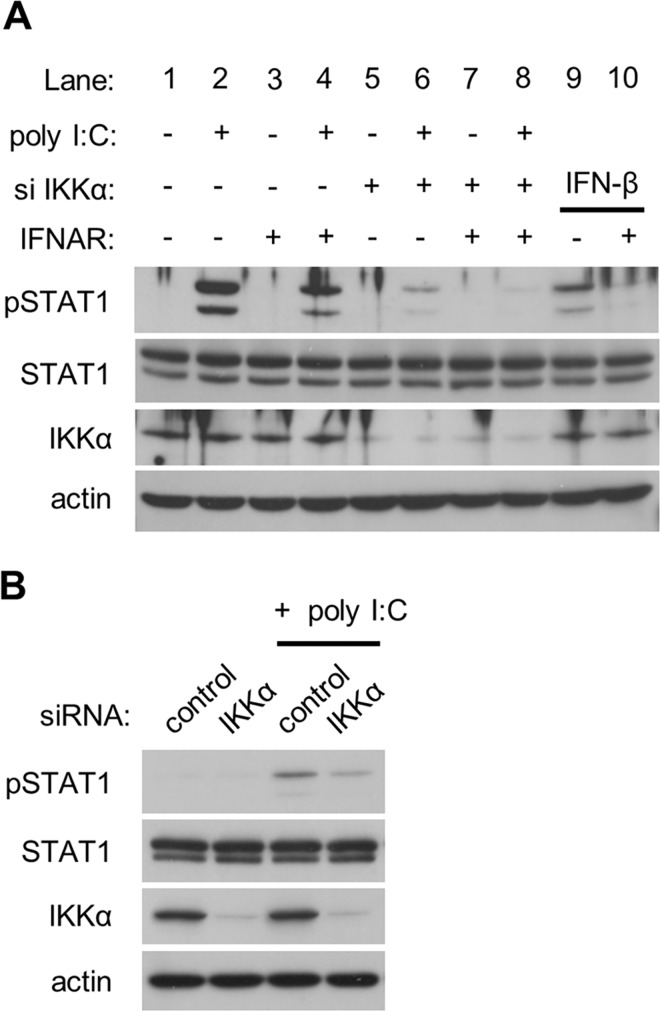
Role of IKKα in type I IFN-dependent and independent STAT1 phosphorylation induced by dsRNA. (A) HeLa cells were transfected with siRNA targeting IKKα or control siRNA for 48 h. Then, the cells were treated with an anti-IFNAR neutralizing antibody for 1 h followed by transfection with poly I:C (50 ng/well) for an additional 4 h. HeLa cells were also treated with IFN-β (200 IU/ml) for 15 min as a control to confirm the neutralizing activity of the anti-IFNAR antibody. (B) IFNAR-null U5A cells were transfected with siRNA targeting IKKα or control siRNA for 48 h, and the cells were then further transfected with poly I:C (500 ng/well) for 10 h. Cell extracts were analyzed by immunoblotting. The results are representative of three independent experiments.

To further confirm whether IKKα is involved in type I IFN-independent STAT1 phosphorylation, we used an anti-type I IFN receptor (IFNAR)-neutralizing antibody to block type I IFN-mediated STAT1 phosphorylation. Pretreatment of the cells with the neutralizing antibody partially inhibited the phosphorylation of STAT1 in response to poly I:C (Fig 4A, lane 4). These results indicate that RLR signaling can activate STAT1 phosphorylation at least partly in a type I IFN-independent manner. We noted that this neutralizing antibody almost completely inhibited the phosphorylation of STAT1 in response to IFN-β, whose concentration was much greater than that produced by poly I:C transfection (Fig 4A, lane 10). Silencing IKKα drastically suppressed the phosphorylation of STAT1 (Fig 4A, lane 6). Furthermore, in the presence of the type I IFNAR-neutralizing antibody, poly I:C failed to induce STAT1 phosphorylation in IKKα-depleted conditions (Fig 4A, lane 8). These results suggest that IKKα mediates both type I IFN-dependent and independent STAT1 phosphorylation in RLR signaling. Introduction of poly I:C in IFNAR-deficient U5A cells resulted in type I IFN-independent STAT1 phosphorylation (Fig 4B), as shown previously [14]. Silencing IKKα suppressed the level of phosphorylated STAT1 in response to poly I:C in U5A cells, indicating the involvement of IKKα in type I IFN-independent STAT1 phosphorylation activated by RLR signaling. In type I IFN signaling, STAT1 and STAT2 heterodimers associate with IRF9 to form the ISGF3 complex that translocates to the nucleus [25]. Then, we confirmed the effect of IKKα on phospho-STAT2 levels. In IFNAR-expressing 2fTGH cells, STAT2 was phosphorylated in response to poly I:C (S2 Fig). IKKα silencing partially suppressed the STAT2 phosphorylation as well as STAT1 phosphorylation. In contrast, no phosphorylation of STAT2 was observed in IFNAR-deficient U5A cells, indicating that activation of STAT2 in response to poly I:C is type I IFN-dependent.

Various cytokines have been shown to directly activate STAT1, and IL6 is one of the best-known cytokines that activates STAT1 [26]. Therefore, we confirmed the involvement of IKKα in IL6 expression by RLR signaling. As expected, transfection with poly I:C induced both mRNA expression and protein secretion of IL6 (S3 Fig). Silencing of IKKα significantly but partially inhibited the induction of IL6, suggesting a partial contribution of IL6 to IKKα-mediated type I IFN-independent STAT1 activation by RLR signaling.

### Effects of IKKα on activation of IRF3 and NFκB in response to dsRNA

**Fig 5:**
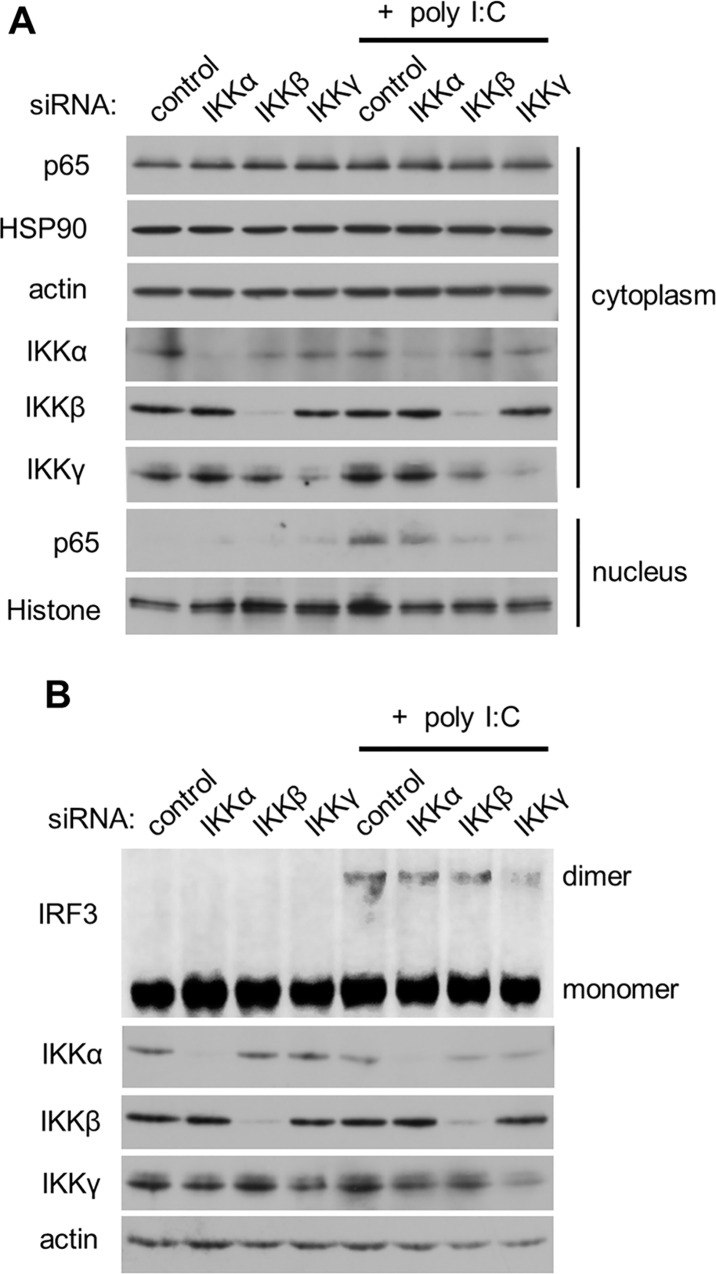
Effects of IKKα on translocation of the NFκB p65 subunit and dimerization of IRF3 in response to dsRNA. IKKα, IKKβ, or IKKγ in HeLa (A) and U5A (B) cells were silenced as described above. Then, the cells were transfected with poly I:C for 4 h. Cell extracts were subjected to SDS-PAGE or native-PAGE (IRF3) followed by immunoblotting. The results are representative of three independent experiments.

Because coordinated activation of NFκB and IRF3 was essential for induction of type I IFN [27], we next investigated the role of IKKα in the activation of NFκB and IRF3 in response to dsRNA. Following activation by poly I:C, NFκB translocated to the nucleus (Fig 5A). Knockdown of IKKβ or IKKγ, but not IKKα, inhibited the translocation of NFκB, suggesting an essential role for IKKβ and IKKγ and a dispensable role for IKKα in RLR-mediated NFκB activation. RLR-mediated IRF3 dimerization was reduced by IKKγ silencing, but knockdown of IKKα or IKKβ had no effect on dimerization (Fig 5B). We further examined the involvement of IKKα in the nuclear translocation of p50, p52, and Rel B, other NFκB subunits. TNF-α, which was used as a positive control, induced the nuclear translocation of p50, p52, and Rel B in HeLa cells (S4 Fig). IKKα silencing partially suppressed the nuclear translocation of p50. Activation of RLR signaling by poly I:C transfection only enhanced the nuclear translocation of Rel B, and IKKα silencing did not inhibit the nuclear localization. We found that knockdown of IKKα enhanced nuclear accumulation of p52 and Rel B by an unknown mechanism. We confirmed that this observation was a non-specific effect by IKKα siRNA because we observed similar results using other IKKα-specific siRNAs (data not shown). These results indicate that IKKα is not involved in canonical RLR signaling, which includes NFκB and IRF3.

### Effects of IKKα on Janus tyrosine kinase phosphorylation

**Fig 6:**
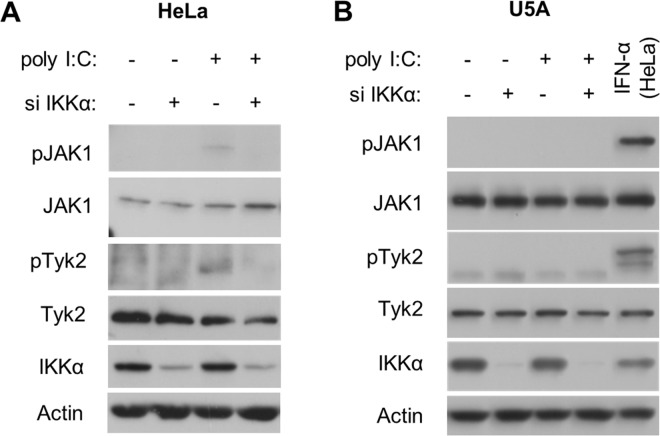
Influence of IKKα knockdown on JAK1 and Tyk2 activation in HeLa and U5A cells. Following knockdown of IKKα in HeLa (A) and U5A cells (B), poly I:C was introduced for an additional 4 h and 10 h, respectively. HeLa cells were treated with IFN-α (10 ng/ml) for 20 min to confirm phosphorylation of JAK1 and Tyk2 as a positive control. Cell extracts were analyzed by immunoblotting. The results are representative of three independent experiments.

Janus tyrosine kinases (JAKs) are upstream of STAT1 in the IFNAR-JAK-STAT axis. We therefore examined whether JAK activation was required for IKKα-mediated STAT1 phosphorylation. Poly I:C induced phosphorylation of JAK1 and Tyk2, both of which serve as kinases for STAT1 [28], in HeLa cells (Fig 6A). Silencing of IKKα suppressed phosphorylation of JAK1 and Tyk2 in response to poly I:C. IFNAR-deficient U5A cells exposed to poly I:C did not show phosphorylation of JAK1 and Tyk2, while poly I:C was able to induce STAT1 phosphorylation in these cells (Fig 4B). These results suggest that 1) activation of JAKs is entirely IFN-dependent; 2) IKKα mediates phosphorylation of JAKs via the expression of type I IFN; and 3) IFNAR-independent STAT1 phosphorylation is IKKα-dependent and JAK-independent.

### No direct interaction between IKKα and STAT1

**Fig 7:**
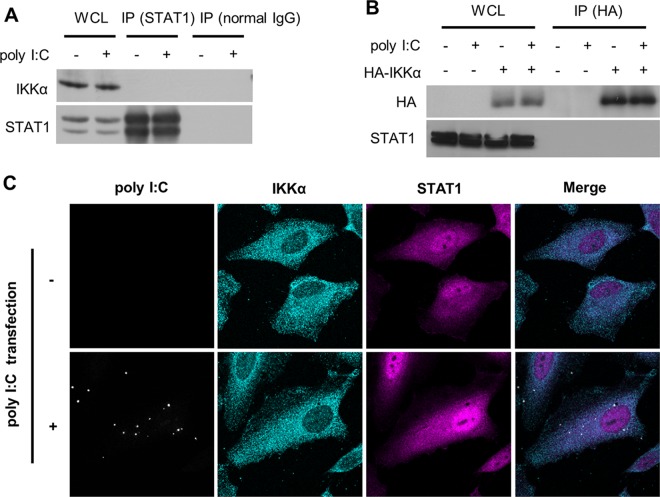
No interaction of IKKα and STAT1. (A) HeLa cells were transfected with poly I:C for 4 h. (B) Twenty-four hours after transfection with a HA-tagged IKKα expression plasmid or an empty plasmid (mock), HeLa cells were transfected with poly I:C for 4 h. Immunoprecipitation (IP) was performed using an anti-STAT1, anti-IgG (A) or anti-HA beads (B), followed by immunoblotting using anti-IKKα, anti-STAT1 or anti-HA antibody. (C) The cells were fixed with 4% paraformaldehyde and incubated with anti-IKKα and anti-STAT1 antibodies. IKKα and STAT1 were then detected separately using a secondary antibody coupled to Alexa 488 (green) or Alexa 555 (red).

We next determined whether IKKα directly regulates STAT1. Immunoprecipitation analysis showed that neither endogenous nor exogenous IKKα interacted with the STAT1 protein in the presence or the absence of poly I:C treatment in the cells (Fig 7A and 7B). In addition, immunofluorescence analyses did not find a physical interaction between IKKα and STAT1, even in poly I:C-transfected cells (Fig 7C).

## Discussion

IKKα has been well-characterized as a regulator of canonical NFκB signaling. In normal conditions, NFκB is sequestered in the cytoplasm by IκB family proteins [16]. The IKK complex consists of IKKα and IKKβ, which function as the catalytic subunits, and IKKγ, which serves as a bridge for the IKKs and interacts with upstream signaling molecules [29]. A variety of stimuli, including cytokines and LPS, induce the IKK complex to undergo a conformational change to phosphorylate IκB, resulting in degradation of IκB and nuclear translocation of NFκB. Although the phosphorylation of specific serine residues in either IKKα or IKKβ can activate the IKK complex, a study revealed that IKKβ alone is sufficient for canonical NFκB activation [30]. A variety of stimuli activate the major NFκB p65 subunit (RelA). NFκB p65 is phosphorylated in response to pro-inflammatory stimuli [31]. IκB degradation and NFκB activation were undetectable in IKKγ-deficient mouse embryonic fibroblasts (MEFs), suggesting an essential role for IKKγ in the classical NFκB pathway [32]. Collectively, IKKβ and IKKγ were shown to be indispensable for canonical NFκB activation, but the role of IKKα is unclear. In the canonical pathway, NFκB p65 is phosphorylated at Ser-276, leading to an increase in its transcriptional activity [33]. In contrast, IKKα could recognize NFκB but not IκB as a substrate and phosphorylated the protein at Ser-536 in response to TNF-α [34]. Indeed, our present study indicated that poly I:C did not alter the phosphorylation of NFκB p65 at Ser-536 (data not shown) or the nuclear translocation of NFκB p65 (Fig 5A). The amino acid sequences of IKKα and IKKβ are structurally similar. However, IKKβ is restricted to the cytosolic domain, whereas IKKα can shuttle from the cytoplasm to the nucleus following stimulation [35]. Our data showed that RLR signaling did not affect the intracellular distribution of IKKα (Fig 7C). These results indicate a complicated and unique role of IKKα in the non-canonical NFκB signaling pathway.

In NFκB signaling, both canonical and alternative pathways were reported to be involved in the innate immune reaction induced by viruses. Viruses have evolved multiple mechanisms to activate or inhibit these pathways to promote replication or to maintain infection, partly by manipulating IKKα. For example, the Molluscum contagiosum virus (MCV) inhibited the NFκB cascade via degradation of IKKα by the viral protein MC160, although MC160 did not interact with IKKα directly [36]. In contrast to MCV, herpes simplex virus (HSV)-1 replication was dependent on the activation of the NFκB cascade. An obvious loss of HSV virus yield and reduced NFκB nuclear translocation were observed in IKKα-deficient MEFs [37]. Additionally, IKKα is involved in hepatitis C virus (HCV)-induced lipogenesis and viral assembly independent of NFκB activation. IKKα was activated by the interaction of DDX3X with the HCV 3' untranslated region and then induced CBP/p300-mediated lipogenic gene transcription, leading to core-associated lipid droplet formation, which facilitates viral assembly [38]. In addition, IKKα was required for functional maturation of dendritic cells and acquired immunity to *Listeria monocytogenes* infection [39]. These findings clearly show the essential NFκB signaling-independent role of IKKα in antiviral innate immunity. In fact, a recent study found that IKKα regulates IRF3-mediated transcription [39].

In this study, we report a role of IKKα in both type I IFN-dependent and independent phosphorylation of STAT1 in response to the activation of RLR signaling. Although we did not identify a role of IKKα in poly I:C-induced IRF3 dimerization, silencing of IKKα markedly inhibited the transcription of IFN-β mRNA in response to poly I:C. These results indicate that IKKα regulates antiviral signaling via an IRF3-independent mechanism. The molecules that are involved in IKKα-mediated antiviral signaling remain to be elucidated. In RLR signaling, few reports have shown involvement of the transcription factor AP-1, which mediates gene regulation of a variety of cytokines [40]. Indeed, AP-1 regulates the expressions of IFN-β [41] and IL6 [42]. A recent study identified a possible interaction of IKKα with AP-1 [43]. This may explain the type I IFN-independent and IKKα-dependent STAT1 activation in RLR signaling. We previously reported that RLR signaling can activate type I IFN-independent STAT1 phosphorylation [14]. Interestingly, activation of the type I IFN-independent STAT1 is unrelated to the expression of ISGs. We also observed that knockdown of IKKα did not alter the level of ISGs in response to poly I:C in IFNAR-deficient cells (data not shown). RLR signaling can induce a variety of cytokines, including CCL5 [44] and IL6 [45]. In the present study, we demonstrated that IL6 can at least partially contribute to type I IFN-independent and IKKα-dependent STAT1 phosphorylation in RLR signaling. Our results using IFNAR-deficient cells showed that dsRNA activates STAT1 with delayed kinetics (8–10 h after dsRNA transfection). This indicates that synthesized proteins, including cytokines, may be required to activate STAT1 in a type I IFN-independent manner. Collectively, these results combined with our previous data suggest that the type I IFN-independent STAT1 phosphorylation is regulated by IKKα, and activated STAT1 may be involved in signaling pathways with functions other than the expression of ISGs.

**Fig 8:**
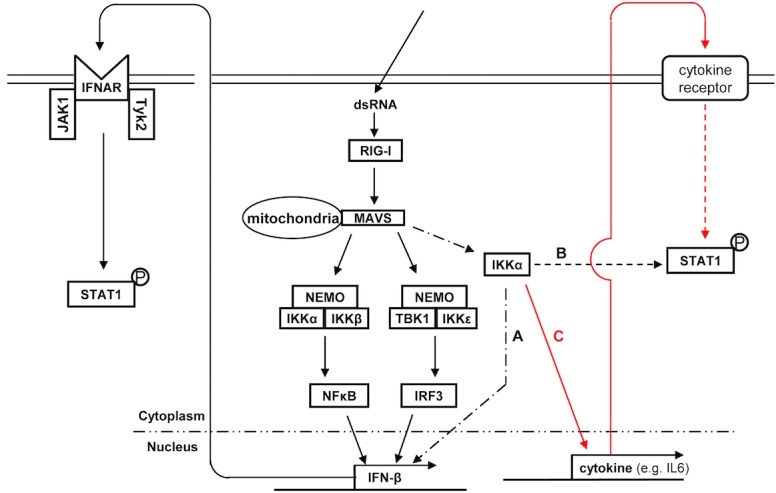
Comprehensive roles of IKKα in RLR signaling-mediated STAT1 activation. In addition to participating in the IKK complex to activate the canonical antiviral signaling pathway, IKKα mediates type I IFN expression (a long dashed dotted line: A). IKKα could directly activate STAT1 phosphorylation in a type I IFN-independent manner (a dashed line: B). IKKα-meditated cytokine expression likely activates STAT1 via a type I IFN-independent positive feedback loop (red line: C).

In summary, we investigated the function of IKKα in RLR signaling (Fig 8). RLR signaling-mediated IKKα did not affect NFκB or IRF3, both of which are known as “classical” antiviral signaling molecules. However, IKKα altered the expression of IFN-β in response to dsRNA; therefore, IKKα induces IFN-β via an unknown non-canonical pathway [Fig 8, a long dashed dotted line (A)]. In this pathway, STAT1 was phosphorylated in a type I IFN-dependent manner. In addition, IKKα also phosphorylated STAT1 in a type I IFN-independent manner [Fig 8, a dashed line (B)]. Cytokines induced by dsRNA can drive a positive feedback loop in type I IFN-independent STAT1 phosphorylation [Fig 8, red line: (C)]. Although these pathways can phosphorylate STAT1 at Tyr-701, the role of type I IFN-independent STAT1 phosphorylation differs from that of type I IFN-dependent STAT1 phosphorylation. This novel role of IKKα in STAT1 phosphorylation may be essential for antiviral innate immunity.

## Supporting Information

S1 Fig Effect of an IKKα inhibitor on IκB degradation by TNFα. HeLa cells were pretreated with an IKK inhibitor at the indicated concentrations for 1 h, and then, cell extracts were analyzed by immunoblotting. The results are representative of three independent experiments.
(TIF)

S2 Fig Effect of IKKα on dsRNA-induced STAT2 phosphorylation. Following knockdown of IKKα in HeLa (A) and U5A cells (B), poly I:C was introduced for an additional 4 h and 10 h, respectively. Cell extracts were analyzed by immunoblotting. The results are representative of three independent experiments.
(TIF)

S3 Fig Involvement of IKKα in IL6 induction in response to dsRNA. Following knockdown of IKKα, HeLa cells were transfected with poly I:C for an additional 48 h (A, B). IL6 mRNA (A) or protein (B) expression was examined. All data are shown as the mean of three independent experiments. *P<0.01.
(TIF)

S4 Fig Effects of IKKα on translocation of NFκB p50, p52, and Rel B subunits and dimerization of IRF3 in response to dsRNA IKKα in HeLa cells was described previously. Then, the cells were transfected with poly I:C for 4 h or stimulated with TNF-α (5 ng/mL) for 3 h. Cell extracts were subjected to SDS-PAGE followed by immunoblotting. CBB staining of the transferred membrane was used as a loading control. The results are representative of three independent experiments.
(TIF)
