# Supplementary figures and images for: Non-Canonical Role of IKKα in the Regulation of STAT1 Phosphorylation in Antiviral Signaling
Source: PLoS One. 2016 Dec 19;11(12):e0168696. doi: 10.1371/journal.pone.0168696 (PMC5167405; doi:10.1371/journal.pone.0168696)

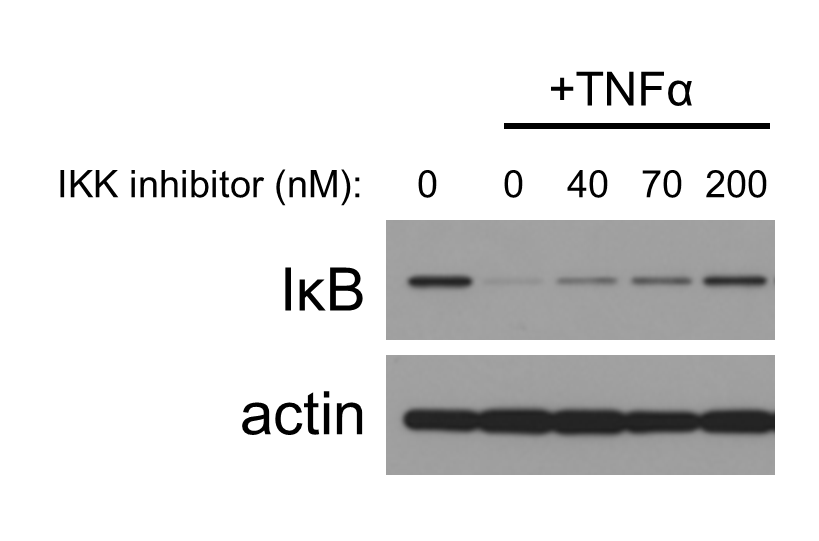

Supplement: S1 Fig — HeLa cells were pretreated with an IKK inhibitor at the indicated concentrations for 1 h, and then, cell extracts were analyzed by immunoblotting. The results are representative of three independent experiments. (TIF) [file pone.0168696.s001.tif]

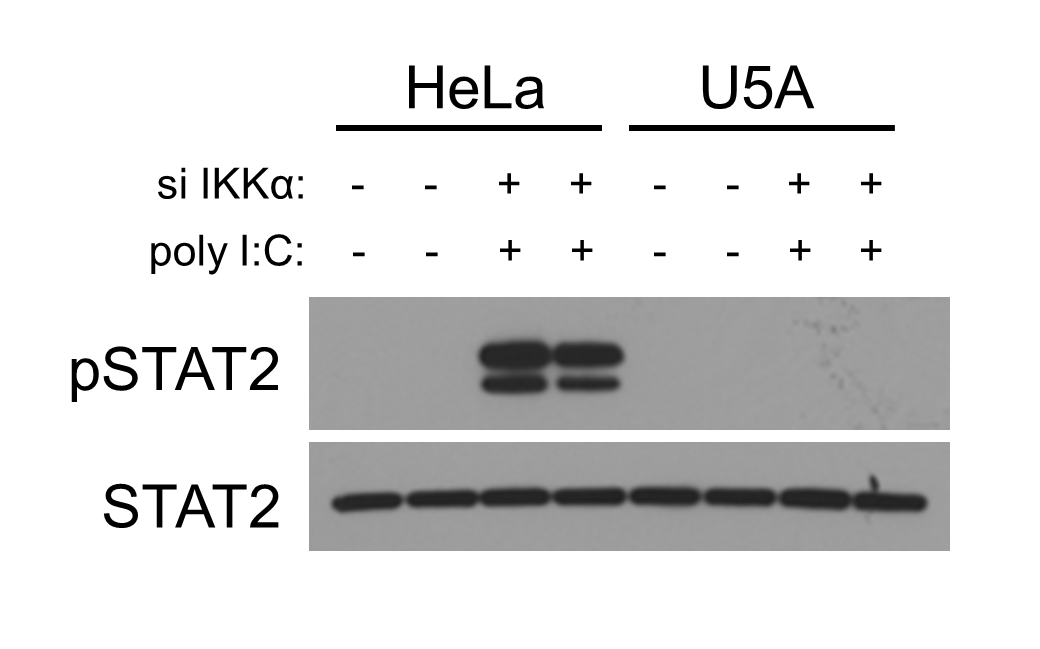

Supplement: S2 Fig — Following knockdown of IKKα in HeLa (A) and U5A cells (B), poly I:C was introduced for an additional 4 h and 10 h, respectively. Cell extracts were analyzed by immunoblotting. The results are representative of three independent experiments. (TIF) [file pone.0168696.s002.tif]

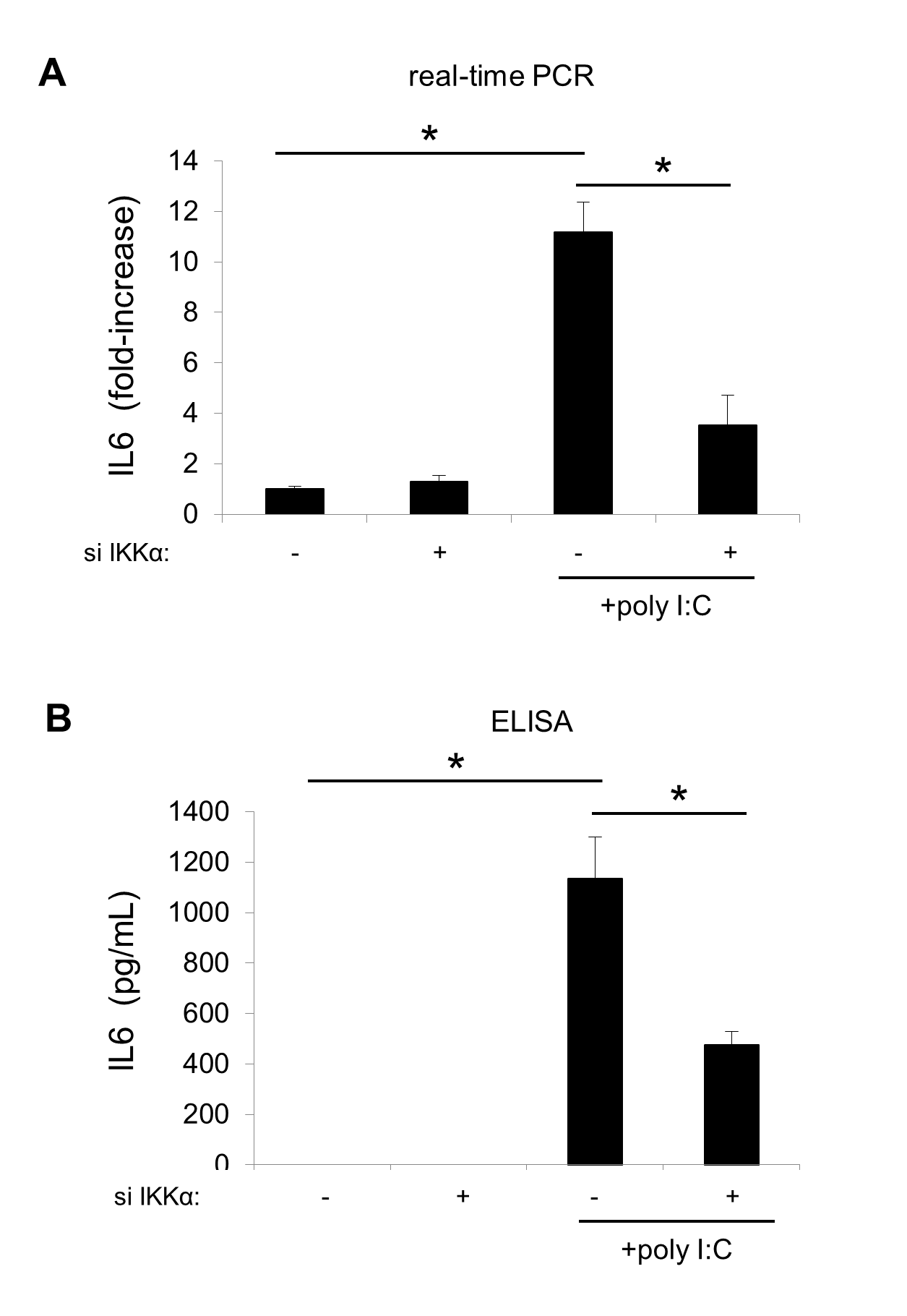

Supplement: S3 Fig — Following knockdown of IKKα, HeLa cells were transfected with poly I:C for an additional 48 h (A, B). IL6 mRNA (A) or protein (B) expression was examined. All data are shown as the mean of three independent experiments. *P<0.01. (TIF) [file pone.0168696.s003.tif]

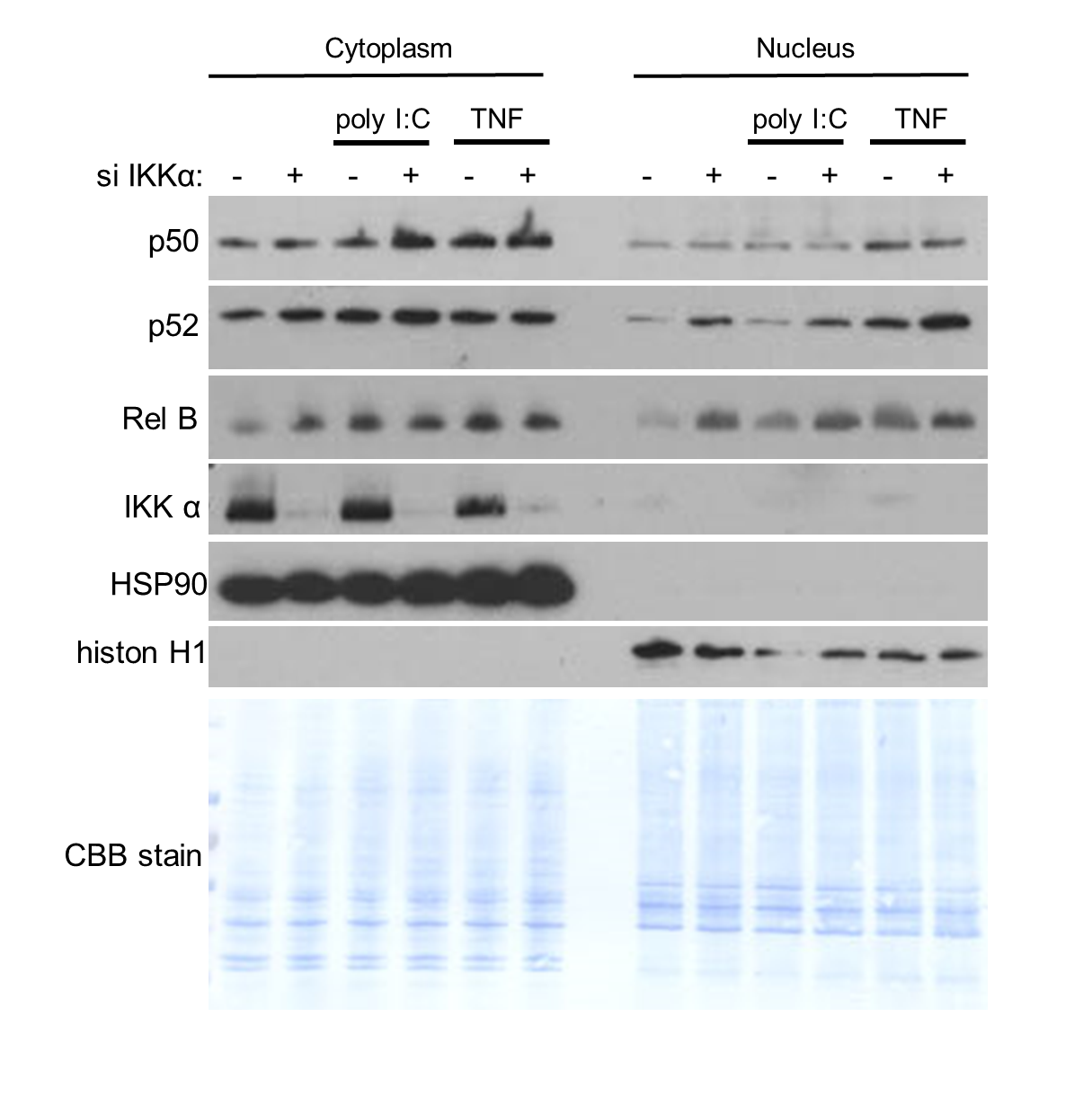

Supplement: S4 Fig — Then, the cells were transfected with poly I:C for 4 h or stimulated with TNF-α (5 ng/mL) for 3 h. Cell extracts were subjected to SDS-PAGE followed by immunoblotting. CBB staining of the transferred membrane was used as a loading control. The results are representative of three independent experiments. (TIF) [file pone.0168696.s004.tif]
